# Supplementary material for: Mitochondrial DNA Variation in Peruvian Honey Bee (Apis mellifera L.) Populations Using the tRNAleu-cox2 Intergenic Region
Source: Insects. 2021 Jul 14;12(7):641. doi: 10.3390/insects12070641 (PMC8303314; doi:10.3390/insects12070641)
Supplement: Supplementary file 1 [file insects-12-00641-s001.zip › insects-1271778-supplementary.pdf]

Table S1. Geographical coordinates for sampling sites in three Peruvian regions.

| Region | Sampling sites | Latitude | Longitude |
|---|---|---|---|
| Lima | Barranca | -10.69414582 | -77.69013187 |
| | Coayllo | -12.73032341 | -76.45966615 |
| | Quilmaná | -12,95545436 | -76,38696181 |
| | Santa María | -11,09434808 | -77,58587021 |
| | Sayán | -11,2340555 | -77,37451224 |
| Piura | Chulucanas 1 | -4,985357156 | -80,16514518 |
| | Chulucanas 2 | -5,120997583 | -80,19520977 |
| | La Matanza 1 | -5,215620746 | -80,0926018 |
| | La Matanza 2 | -5,371220445 | -80,02215688 |
| | Piura | -5,175398077 | -80,7074544 |
| Junín | La Merced | -11,0703574 | -75,33402035 |
| | Perené 1 | -10,9023300 | -75,19011 |
| | Perené 2 | -10,99490867 | -75,20002374 |
| | San Ramón | -11,15000226 | -75,38637896 |
| | Vitoc | -11,21033800 | -75,33533191 |

Table S2. Haplotype frequencies and sample sizes (on the third line) for each sampling site from Lima, Piura, and Junín.

| | **Lima** | | | | | **Piura** | | | | | **Junín** | | | | |
|---|---|---|---|---|---|---|---|---|---|---|---|---|---|---|---|
| | **Barranca** | **Coayllo** | **Quilmaná** | **Santa María** | **Sayán** | **Chulucanas 1** | **Chulucanas 2** | **La Matanza 1** | **La Matanza 2** | **Piura** | **La Merced** | **Perené 1** | **Perené 2** | **San Ramón** | **Vitoc** |
| **Haplotype** | **42** | **38** | **36** | **39** | **35** | **38** | **31** | **14** | **52** | **31** | **32** | **36** | **29** | **35** | **24** |
| **C1** | 0.905 | - | 0.194 | 0.641 | 0.257 | - | 0.258 | 0.071 | 0.096 | - | - | - | - | - | - |
| **C2** | - | - | 0.056 | - | - | - | - | - | - | 0.032 | - | - | - | - | - |
| **C2c** | 0.048 | - | - | 0.205 | - | - | - | - | - | - | - | - | - | - | - |
| **C2j** | - | 0.632 | - | 0.051 | 0.314 | - | 0.065 | - | - | - | 0.25 | 0.028 | 0.069 | 0.143 | 0.208 |
| **C2l** | - | - | - | - | - | 0.079 | - | - | - | - | - | - | - | - | - |
| **C3** | - | - | 0.111 | - | - | - | - | - | - | - | - | - | - | - | - |
| **M7b** | - | - | 0.056 | - | - | - | - | - | - | - | - | 0.028 | - | - | 0.083 |
| **M7c** | - | - | - | - | - | - | - | - | - | 0.032 | - | - | - | - | - |
| **A30** | - | 0.342 | 0.111 | 0.077 | 0.029 | 0.421 | - | 0.214 | 0.192 | 0.032 | - | - | - | - | - |
| **A30d** | - | - | 0.028 | - | - | - | - | - | - | - | - | - | - | - | - |
| **A30e** | - | - | 0.028 | - | - | - | - | - | - | - | - | - | - | - | - |
| **A4p** | - | 0.026 | - | 0.026 | 0.286 | 0.447 | 0.452 | 0.214 | 0.327 | 0.452 | 0.188 | 0.278 | 0.172 | 0.2 | 0.5 |
| **A4q** | - | - | - | - | - | 0.026 | - | - | - | - | - | - | - | - | - |
| **A4s** | - | - | - | - | - | - | - | - | - | - | 0.031 | - | - | - | - |
| **A4t** | 0.048 | - | 0.361 | - | - | - | 0.161 | 0.143 | 0.192 | 0.258 | 0.344 | 0.583 | 0.586 | 0.057 | 0.208 |
| **A4u** | - | - | - | - | - | - | - | - | - | - | - | 0.028 | 0.069 | 0.029 | - |
| **A4v** | - | - | - | - | - | - | - | - | - | - | - | - | 0.034 | - | - |
| **A4w** | - | - | - | - | - | - | - | - | - | - | - | - | 0.069 | - | - |
| **A1** | - | - | - | - | - | - | 0.032 | 0.214 | 0.077 | 0.097 | 0.031 | - | - | 0.257 | - |

| | | | | | | | | | | | | | | | |
|---|---|---|---|---|---|---|---|---|---|---|---|---|---|---|---|
| **A1e** | - | - | - | - | 0.114 | 0.026 | - | 0.143 | 0.115 | 0.065 | 0.063 | 0.028 | - | 0.086 | - |
| **A1t** | - | - | 0.056 | - | - | - | - | - | - | - | - | - | - | - | - |
| **A1u** | - | - | - | - | - | - | - | - | - | - | 0.063 | - | - | 0.229 | - |
| **A1w** | - | - | - | - | - | - | - | - | - | - | - | 0.028 | - | - | - |
| **A65** | - | - | - | - | - | - | 0.032 | - | - | 0.032 | 0.031 | - | - | - | - |
